# The comparisons of phenotype and genotype between CADASIL and CADASIL-like patients and population-specific evaluation of CADASIL scale in China

**DOI:** 10.1186/s10194-016-0646-5

**Published:** 2016-05-20

**Authors:** Dan He, Daiqi Chen, Xuefei Li, Zheng Hu, Zhiyuan Yu, Wei Wang, Xiang luo

**Affiliations:** Department of Neurology, Tongji Hospital, Tongji Medical College, Huazhong University of Science and Technology, 1095 Jiefang Avenue, Wuhan, Hubei 430030 China; Department of Neurology, The first affiliated hospital, Sun Yat-sen University, Guangzhou, Guangdong China; Department of Obstetrics and Gynecology, The first affiliated hospital, Sun Yat-sen University, Guangzhou, Guangdong China

**Keywords:** CADASIL, Small vessel disease, Phenotype, Genotype, CADASIL scale

## Abstract

**Background:**

Cerebral autosomal dominant arteriopathy with subcortical infarcts and leukoencephalopathy (CADASIL) is the most common form of hereditary stroke disorder caused by mutations in the *NOTCH3* gene. Although CADASIL scale is a widely used tool to screen clinically suspected CADASIL patients, the differential effects of this scale in various populations remain unknown.

**Methods:**

92 CADASIL-like patients and 24 CADASIL patients were selected based on CADASIL scale and gene tests. The clinical, genetic and radiological characteristics were analyzed.

**Results:**

Based on the CADASIL scale, we first screened 116 suspected CADASIL patients, and detected 20 mutations in 24 CADASIL-patients (Specificity: 20.69 %). Surprisingly, we found that transient ischemic attack/stroke, migraine, cognitive decline, psychiatric disturbances and early onset age in CADASIL scale showed no differences between the CADASIL and the CADASIL-like patients (*p* > 0.05). Instead, recurrent cerebral ischemic events (58.33 %, *p* = 0.028) and positive family histories (*p* < 0.05) were more frequently observed in CADASIL patients. Moreover, compared with CADASIL-like patients (21.74 %), CADASIL patients demonstrated higher percentage of temporal pole involvements (58.33 %, *p* = 0.001), but not the external capsule involvements (66.67 %, *p* = 0.602), in MRI imaging. Further, we found that vascular risk factors could occur in both CADASIL patients and CADASIL-like patients, and therefore could not be used as the markers to differentiate the two groups in our study (*p* > 0.05). By performing DSA analysis, we for the first time identified dysplasia of cerebral blood vessels in CADASIL patients, which were detected more frequently in CADASIL patients (41.67 %) in comparison with CADASIL-like patients (8.69 %, *p* <0.01).

**Conclusion:**

Our data suggested that the efficacy of CADASIL scale to diagnose the disease varied with specific populations. Recurrent cerebral ischemic events, temporal pole involvements (but not the external capsule) in MRI imaging and dysplasia of cerebral blood vessels in DSA may be the new potential risk factors of the CADASIL scale suitable for Chinese patients. Gene testing by encephalopathy gene panel is expected to improve the accuracy of CADASIL differential diagnosis and increase the understanding of this disease in the future.

## Background

Cerebral autosomal dominant arteriopathy with subcortical infarcts and leukoencephalopathy (CADASIL) is a dominantly hereditary small-vessel disease caused by mutations of the *NOTCH3* gene in 19p13 [1, 2]. These mutations occur mostly in the exons 2–24 of the NOTCH3 gene encoding for the epidermal growth factor-like (EGF-like) repeats, and lead to an odd number of cysteine residues with EGF-like region [2, 3]. This disease is clinically characterized by migraine, recurrent cerebrovascular events, psychiatric disturbance, and cognitive impairment that eventually leading to dementia and disability [1, 4]. The most typical pathological features of CADASIL is the accumulation of granular osmiophilic material (GOM) in the walls of small arteries on ultrastructural examination [5]. In addition, CADASIL patients usually demonstrated diffuse white-matter changes in deep white matter, external capsules and anterior pole of temporal lobes in the MRI imagings [6–8].

The diagnosis of CADASIL is suspected when the patients showed typical clinical manifestations and demonstrated diffuse white matter changes that frequently extend to the temporal pole or lacunar infarcts in MRI imaging. Unfortunately, although CADASIL is a single-gene disorder of cerebral small vessel, the clinical characteristics and neuroimaging manifestation varies even within families [9, 10], increasing the difficulty in recognizing the probands of CADASIL. GOM detection has been considered as a more specific method to diagnose CADASIL [4]. However, the inconsistent sensitivities reported by different research groups [4, 11, 12] restricted the usage of GOM detection to confirm the diagnosis of CADASIL. Up till now, gene mutation analysis of *NOTCH3* still remains the golden standard to diagnose the genetically inherited disease. And there are more than 230 different mutations located in 20 different exons reported in CADASIL patients [4, 13].

Nevertheless, *NOTCH3* gene mutation analysis is still costly and time-consuming [4, 14]. Therefore, it is reasonable to pre-screen the suspected patients first to avoid unnecessary gene mutation tests. The most frequently used screening tool is the CADASIL scale established by Francesca Pescini et.al. [15]. This scale was reported to demonstrate a sensitivity of 96.7 and a specificity of 74.2 % in a pooled analysis of patients from different populations. However, with the increasing identification of CADASIL worldwide [14, 16–18], accumulating evidences from different studies suggested the clinical and genomic characteristics of CADASIL varied extensively among different regions [1, 2, 10, 14, 19–21]. Thus, it is necessary to further testify the available CADASIL scale on more patients from different regions, thereby evaluating the clinical efficacy of this scale in various populations. In this study, to contribute to the more specific characterizations of the genotype and the phenotype of CADASIL, and more importantly, to refine the pre-genetic screening tool, we investigated the clinical manifestations, vascular risk factors, neuroimaging characteristics, skin biopsies and *NOTCH3* gene mutation spectrums among 116 Chinese patients from Tongji hospital in WuHan, China. Furthermore, we compared the data between CADASIL and CADASIL-like patients to identify new potential risk factor of CADASIL scale suitable for Chinese patients.

## Methods

### Patients

From January 2009 to December 2014, we performed NOTCH3 gene analysis in 144 inpatients who were suspected with CADASIL at Tongji Hospital in China. Then we retrospectively analysis the scores of CADASIL scale and recruited 116 patients with a CADASIL scale score ≥ 8 [6]. All studies were approved by the local ethics committee, and informed consents were obtained from all the participants. 24 of 116 patients were genetically confirmed to be diagnosed as CADASIL with *NOTCH3* mutation [3]. At the mean time, 92 patients who had no mutation were grouped as CADASIL-like patients.

### Clinical assessment

Specific clinical data of CADASIL were recorded in detail including sex, age, age at onset, age at diagnosis, onset-symptoms, family histories and the history of presence of vascular risk factors (including elevated blood pressure, cigarette smoking, alcoholic intake, diabetes mellitus, hyperhomocysteinanemia, heart disease). Stroke and transient ischemic attack (TIA) were diagnosed according to the standard critiria [22]. Migraine were classified according to the International Classification Headache Disorders [23, 24]. Cognitive decline and psychiatric disturbance were recorded if previous diagnosis has been made by a physician or if the patients exists cognitive decline, mood or behavior disorder referred by themselves or their families. All patients underwent magnetic resonance scans including T1, T2, Flair inversion. Electron microscopy (EM) examination for GOM by skin biopsy were performed in 14 of 24 CADASIL patients. The serum level of glutamic-pyruvic transaminase (ALT), glutamic oxalacetic transaminase (AST), cholesterol (Chol), triglyceride (TG), high-density lipoprotein(HDL), low-density lipoprotein (LDL), homocysteine, creatinine (Cr),N-terminal-brain natriuretic peptide (NT-proBNP), cardical troponin-I (cTnI), and fasting blood-glucose (FBG)were tested. The peripheral blood vessels were examined by color Doppler Ultrasoud, whlile the blood vessels of neck and head were asessed by comupted tomography angiopraphy (CTA) or digital subtraction angiography (DSA). The average diameter of the major blood vessels of the brain were mesuared. When the artery of one side was signifcant thinner than that of the other side, it is termed as dysplasia.

### Genetic study

Genomic DNA was extracted from peripheral blood samples using blood DNA extraction kit according to the manufacturer’s recommendations (Qigen, Germany). Polymerase chain reaction (PCR) was performed with primers (comprising intron–exon boundaries) specific for exons 3, 4, 11, 18 of the *NOTCH3* gene firstly. Following purification of PCR products, sequencing was performed using the automated sequencer ABI 3730 (Applied Biosystems, Foster City, CA, USA). If no mutations were present in these exons, the remaining exons were also analysed. Genetic analysis was performed at the Department of Cardiology, Tongji Hospital, China.

### Statistical analysis

Statistical analyses were performed using SPSS version 15.0 (SPSS, Inc., Chicago, Ill., USA). The results are presented as means ± standard error of the mean. Statistical analyses were performed with the unpaired *t*-test, Fisher’s test or Mantel–Haenszel chi-square statistic test. A *P*-value of <0.05 was taken as significant.

## Results

### Diagnosis of CADASIL and the mutation spectrum of *NOTCH3* gene

According to the CADASIL scale, we screened out 116 clinically suspected patients at Tongji Hospital from January 2009 to December 2014. Among these patients, we identified 92 *NOTCH3* mutation negative subjects (grouped as CADASIL-like patients, 40 men and 52 women with a mean age at diagnosis of 57.83 ± 0.85 years), and 24 *NOTCH3* mutation positive subjects (grouped as CADASIL patients, 16 men and 8 women with a mean age at the diagnosis of 54.67 ± 2.50 years), from 24 different and unrelated families.

**Fig. 1:**
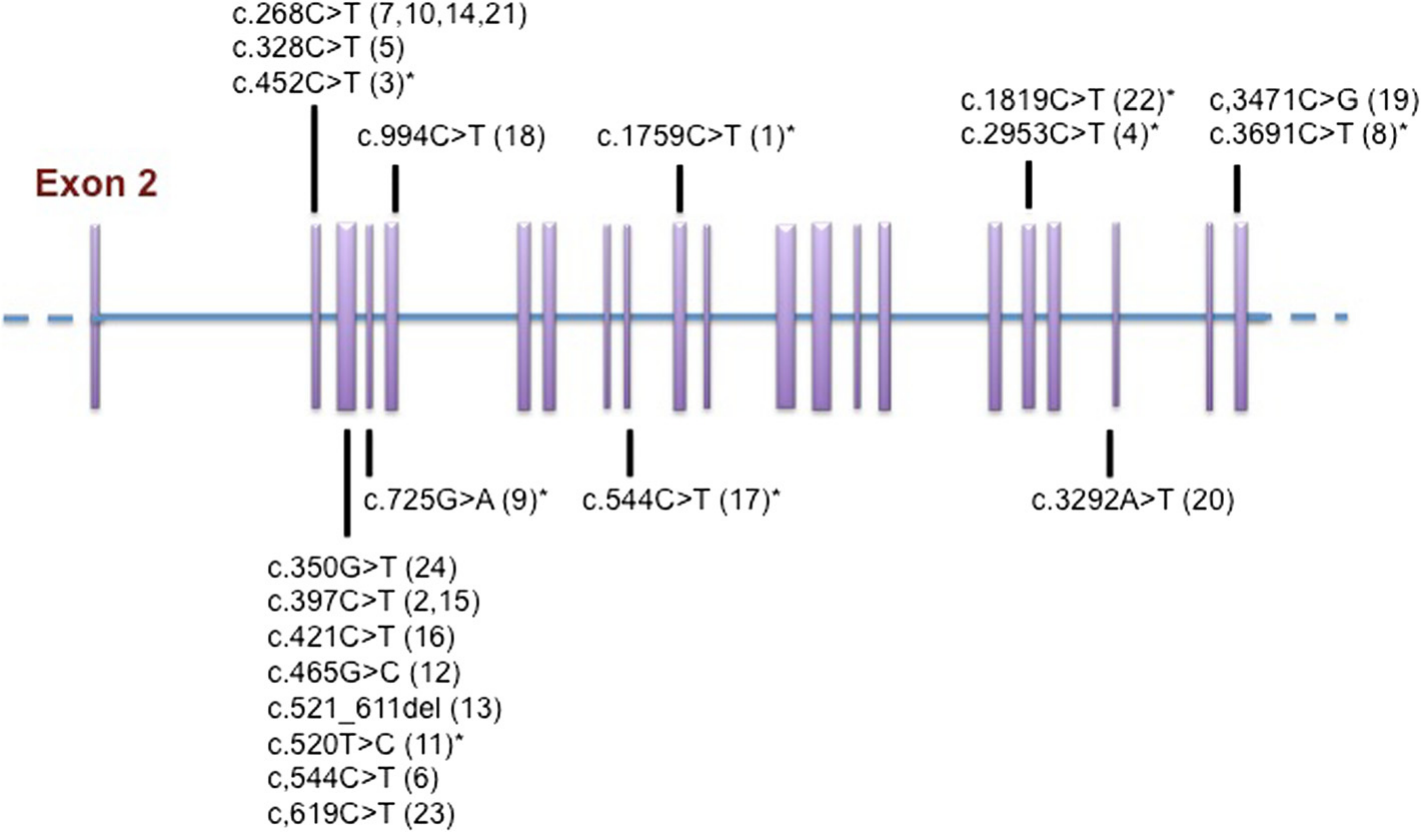
Schematic structure of the NOTCH3 gene mutations in the patients with CADASIL. The mutations which were not reported in previous studies in the mainland of China are marked with asterisks

Our genetic analysis revealed 19 different missense mutations and 1 deletion mutation in these patients, 12 of which have been reported in other researches conducted in China [10, 14, 25], while 8 of which were not (Fig. 1). All of these mutation are heterozygous. *NOTCH3* gene mutations were detected at the exon 4 in 9 out of 24 of our patients (37.5), and at the exon 3 in 6 patients (25 %). The other mutations were located at exon 5 of patient No. 9, exon 6 of patient No. 18, exon 10 of patient No.17, exon 11 of patient No. 1, exon 18 of patient No. 4 and No. 22, exon 20 of patient No. 20 and exon 22 of patient No. 8 and No.19. The majority of mutations in our study were located in exon 4 and 3, followed by exon 18 and 22. But for CADASIL patients in Europe, *NOTCH3* mutations commonly occur in exon 4, followed by exon 3, 5, and 6 [3, 18, 26, 27]. In addition, of the above mutations, Notch 3 mutation of p.Arg90Cys occured recurrently (4 of 24 CADASIL patients) in our study, which was not detected in Caucasian population [13].

**Fig. 2:**
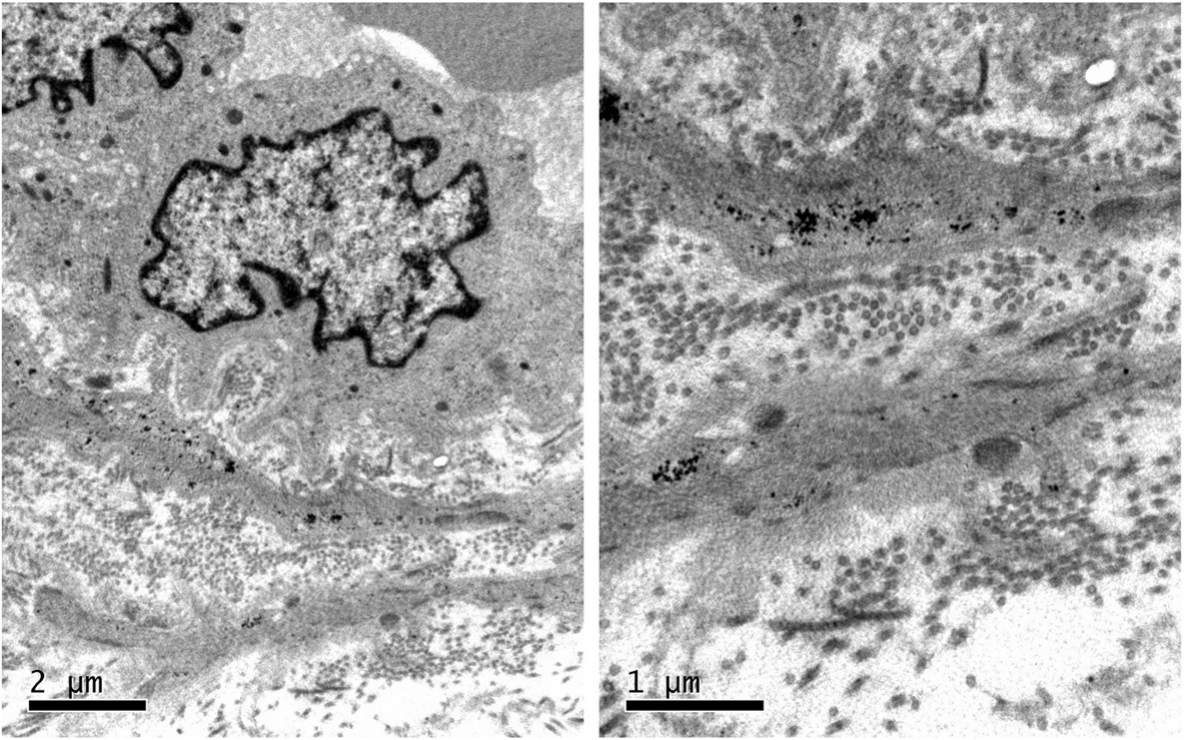
A typical image of GOM detection by skin biopsy from a 42-year-old patient with NOTCH3 p.Arg153Cys

Skin biopsies were taken from 14/24 CADASIL patients to further confirm the diagnosis of CADASIL, and GOM accumulation was detected in the basal layer of vascular smooth muscle cells in 6/14 patients (Fig. 2).

### Comparison of clinical manifestations between CADASIL and CADASIL-like patients

Since CADASIL is often misdiagnosed due to the non-specific clinical and radiological manifestations, it is necessary to clarify the different phenotypes between CADASIL patients and CADASIL-like patients. In this study, the clinical characteristics of CADASIL and CADASIL-like patients were compared.

In accordance with published data, as shown in Table 1, the mean onset ages were similar between the CADASIL group (52.67 ± 2.64 years old) and CADASIL-like group (55.48 ± 0.88 years old, *p* = 0.200). However, compared with CADASIL-like patients, CADASIL patients demonstrated higher percentage of positive family histories (migraine and acute cerebral events) with autosomal dominant inheritance (Table 1). In addition, CADASIL patients showed higher percentage of recurrent ischemic events than CADASIL-like patients (*p* = 0.028). The scores of CADASIL scales are statistically higher in CADASIL group than CADASIL-like group (*p* = 0.047).

**Table 1:** Clinical features of CADASIL and CADASIL-like patients

Clinical manifestations	CADASIL patients (*n* = 24)	CADASIL-like patients (*n* = 92)	P
Age, years	55.08 ± 2.40	58.89 ± 0.86	0.072
Age at onset	52.67 ± 2.64	55.48 ± 0.88	0.200
Duration from first onset to diagnosis	2.00 ± 0.55	1.07 ± 0.25	0.104
History of vascular risk factors			
Elevated blood pressure, %	33.33	76.09	0.000
Diabettes Mellitus, %	25.00	23.91	0.912
Cigarette smoking, %	58.33	47.83	0.359
Alcohol intake, %	25.00	23.91	0.912
Onset-manifestation			
Migraine, %	33.33	23.91	0.348
acute cerebral events, %	66.67	58.70	0.477
psychiatric disturbances, %	25.00	13.04	0.261
cognitive impairment,%	41.67	41.30	0.974
Recurrent stroke, %	58.33	19.57	0.028
Presence of family history of migraine in at least 1 generation, %	25.00	4.35	0.005
Presence of family history of migrane in at least 2 generation, %	16.66	3.26	0.048
Presence of family history of acute cerebral events in at least 1 generation, %	41.66	8.70	0.000
Presence of family history of acute cerebral events in at least 2 generation, %	33.33	5.43	0.000
Presence of family history of psychiatric disturbance/cognitive impairment at least 1 generation, %	8.30	n.r	-
Presence of family history of psychiatric disturbance/cognitive impairment in at least 2 generation, %	n.r	n.r	-
CADASIL scale	16.2 ± 3.1 (10–22)	14.7 ± 2.5 (8–22)	0.047

CADASIL indicates cerebral autosomal-dominant arteriopathy with subcortical infarcts and leukoencephalopathy; ICH intracranial hemorrhage; n.r, not reported

On the other hand, inconsistent with published data [15], the most common four onset symptoms of CADASIL scale were similar between the CADASIL and the CADASIL-like patients in our study (Table 1, *p* > 0.05), suggesting that these four common onset symptoms only may not be good risk factors of CADASIL scale for Chinese patients.

### Comparison of Neuroimaging data between CADASIL and CADASIL-like patients

**Fig. 3:**
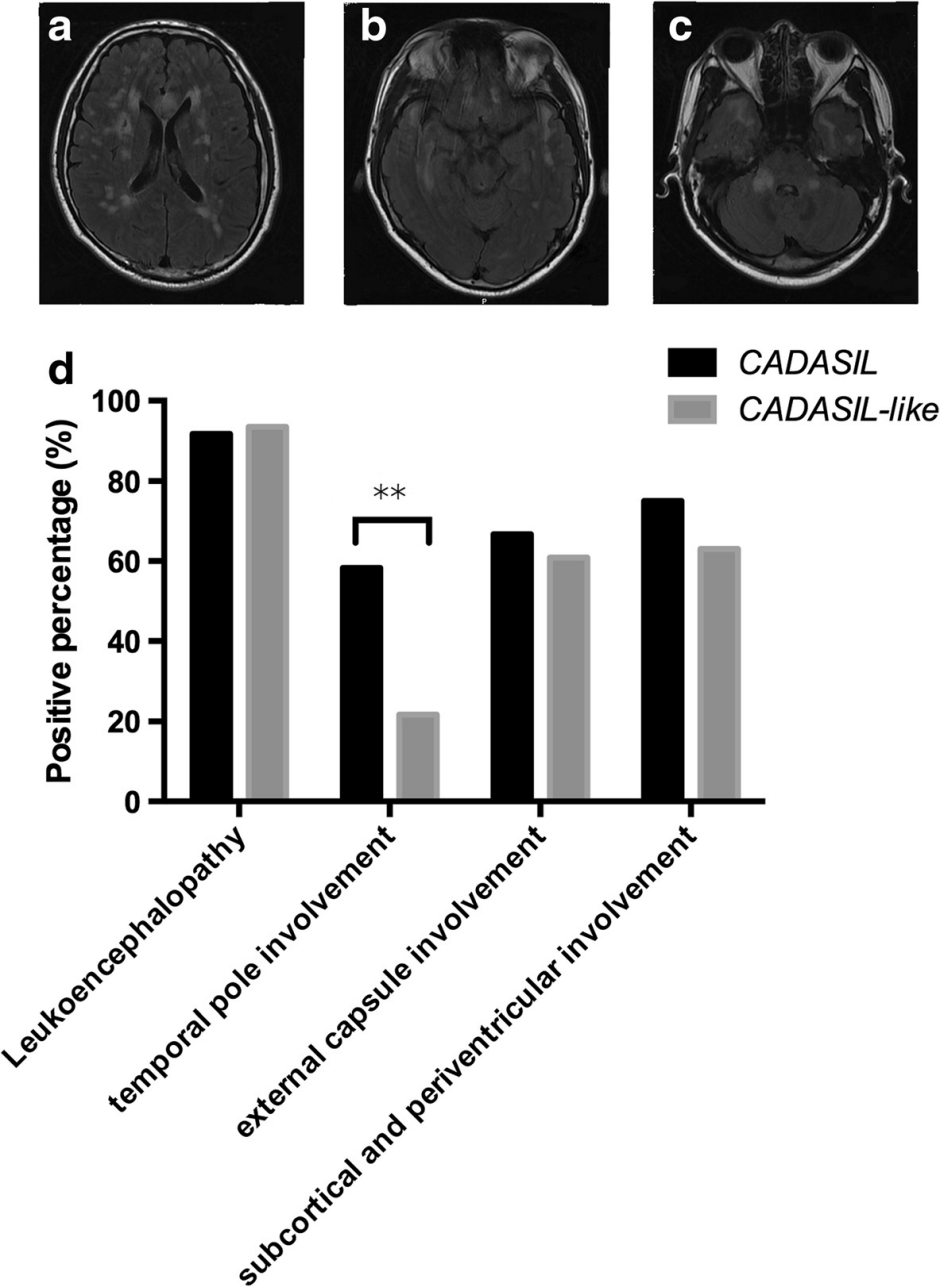
T2-Flair magnetic resonance images from the same 42-year-old patient with NOTCH3 p.Arg153Cys showing diffuse white matter hyperintensities in (**a**) bilateral centrum semiovale, (**b**) temporal pole and (**c**) pedunculus cerebellaris medius. (**d**) indicated the percentage of the positions involved (**p < 0.01)

To further testify the suitability of CADASIL scale, we analyzed the typical neuroimaging features of CADASIL patients (Fig. 3). The comparison of MRI scans showed that the presence of leukoencephalopathy (91.67 % for CADASIL group and 93.48 % for CADASIL-like group, *p* = 0.760), subcortical and periventricular white matter (75 for CADASIL group and 63.04 % for CADASIL-like group, *p* = 0.272), capsula extrema involvement (66.67 for CADASIL group and 60.87 % for CADASIL-like group, *p* = 0.602) were similar between two groups (Fig. 3), which were consistent with previous report. However, temporal pole involvement was significantly more common in CADASIL patients (58.33 %) than in CADASIL-like patients (21.74 %, *p* = 0.001), indicating that temporal involvement may be a higher-risk factor for CADASIL patients in our study.

### Comparison of risk factors for cerebral vascular diseases in CADASIL and CADASIL-like patients

Traditionally, it is believed that the pathological changes of CADASIL are due to the mutations of *NOTCH3* gene, and the development of the CADASIL is not related to other vascular disease such as atherosclerosis. Since both of CADASIL patients and CADASIL-like patients have similar clinical manifestations and diffused leukoencephalopathy in this study, it is necessary to investigate whether cerebral-vascular risk factors could be used as surrogate markers to differentiate CADASIL-like patients (i.e., who may be prone to be related to atherosclerosis) from CADASIL patients.

First, we investigated the common risk factors for cerebral vascular disease in the natural history of these patients. As is shown in Table 1, except for the rate of elevated blood pressure in CADASIL patients (33.33 %) was lower than that in CADASIL-like patients (76.09 %, *p* < 0.01), rates of diabetes, cigarette smoking and alcoholic intake showed no significant difference between the two groups (*p* > 0.05, Table 1).

Second, laboratory tests relating to vascular disease (the serum levels of AST, ALT, LDL, HDL, Cholesterol, TG, homocysteine, proBNP, CTn-I, Cr and FBG) in CADASIL and CADASIL-like patients were also analyzed. Interestingly, hyperlipoproteinemia (33.33 % for CADASIL patients, 36.96 % for CADASIL like patients), hyperhomocysteinemia (58.33 % for CADASIL patients, 67.39 % for CADASIL like patients), decreased HDL (41.66 % for CADASIL patients, 65.21 % for CADASIL like patients) and increased FBG (25 % for CADASIL patients, 28.26 % for CADASIL like patients) were observed in both groups. Except for homocysteine (*p* = 0.043), all the other items displayed similar levels between CADASIL patients and CADASIL-like patients (*p* > 0.05, Table 2).

**Table 2:** Evaluation of vascular risk factors

Laboratory tests	CADASIL patients (*n* = 24)	CADASIL-like patients (*n* = 92)	*P* value
ALT, u/l	26.67 ± 4.96	27.16 ± 4.66	0.964
AST, u/l	25.70 ± 4.90	25.80 ± 2.36	0.986
Chol, mmol/l	3.48 ± 0.34	3.80 ± 0.13	0.330
TG, mmol/l	1.49 ± 0.094	1.50 ± 0.309	0.958
HDL, mmol/l	1.03 ± 0.094	0.99 ± 0.048	0.763
LDL, mmol/l	1.91 ± 0.253	2.40 ± 0.120	0.101
Cr, umol/l	85.38 ± 6.904	86.16 ± 6.577	0.959
homocysteine, umol/l	14.43 ± 1.947	18.8 ± 1.785	0.043
NT-proBNP, pg/ml	252.50 ± 185.114	406.79 ± 204.656	0.706
cTnI, ng/ml	0.02 ± 0.016	0.28 ± 0.275	0.623
FBG, mmol/l	5.85 ± 0.541	6.43 ± 0.467	0.640

**Fig. 4:**
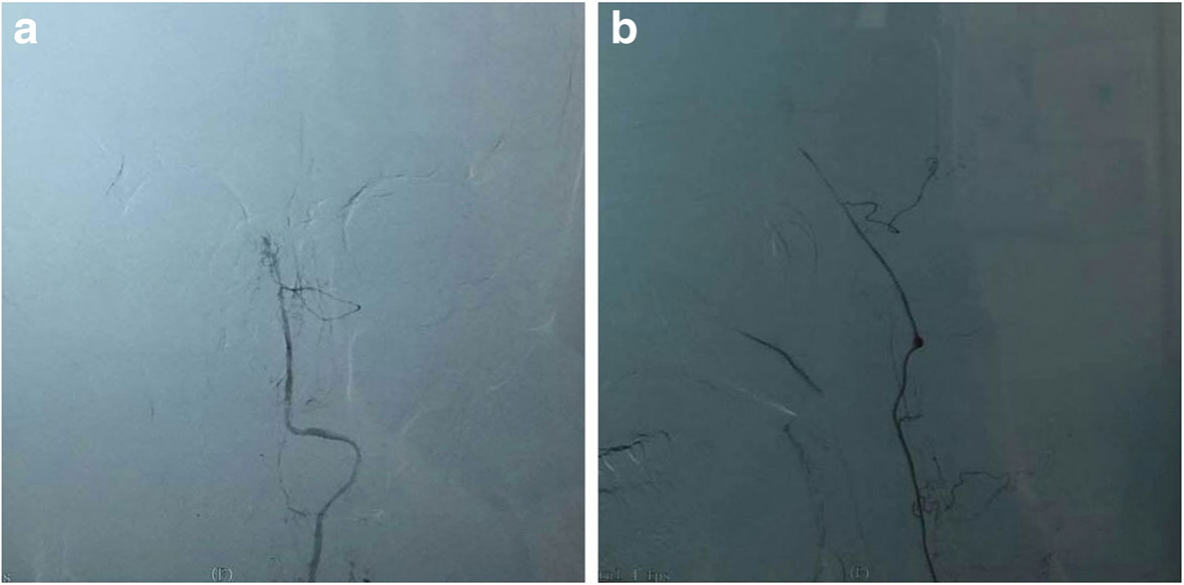
DSA image from the same 42-year-old patient with NOTCH3 p.Arg153Cys. showing angiodysplasia of left vertebral artery on the (**a**) posteroanterior and (**b**) lateral view

Further, both cerebral blood-vessels and the peripheral blood-vessels were also assessed by Doppler Ultrasound, DSA or CTA. Intracranial atherosclerosis was unexpectedly found in both CADASIL patients (12/24) and CADASIL-like patients (64/92, *p* = 0.073), while occlusion of cerebral arteries occurred in 2 CADASIL-like patients. Of note, by performing DSA, we for the first time found that CADASIL patients showed a higher frequency of dysplasia of cerebral blood vessels (which is defined as thinner than the blood vessels of the other side) than CADASIL-like patients (Fig. 4, 10/24 in CADASIL patients and 8/92 in CADASIL-like patients, *p* = 0.000). Together, our data suggest that vascular risk factors could occur in both CADASIL patients and CADASIL-like patients and therefore could not be used as markers to differentiate the two groups in our study. Moreover, the existence of vascular risk factors and abnormality of intracranial arteries could not be used as evidences to excluded the diagnosis of CADASIL.

## Discussion

Genetic analysis of NOTCH3 gene remains as the golden standard to diagnose CADASIL. However, due to the heterogeneity of clinical manifestation and the non-pathognomonic pattern of neuroimaging data, recognition of the disease before the genetic test is challenging. As a pregenetic screening tool, the CADASIL scale based on patients’ clinical characteristics and neuroimaging data was recently developed to narrow down the potential targets for further genetic evaluations [15, 28]. In our study, we recruited 116 clinical suspected CADASIL patients based on this scale in Tongji Hospital. Surprisingly, only 24/116 (20.69 %) of them were genetically confirmed as CADASIL and grouped as CADASIL patients, while other 92/116 (79.31 %) were grouped as CADASIL-like patients. The prediction rate in our study was not only lower than that based on Caucasian patients, but also lower than the rate reported by other researches in China [10, 14, 29], who did not use this scale for the diagnosis of CADASIL. The data suggested that the current CADASIL scale was needed to be further tested and refined, at least in our Chinese patients. Here, to identify new potential risk factor for the refined CADASIL scale, we compared the clinical manifestations and the neuroimaging data between CADASIL and CADASIL-like patients and analyzed the differences between them.

To our knowledge, our data for the first time demonstrated that recurrent ischemic events, positive family history and temporal pore involvement in MRI could be used to further discriminate the CADASIL patients from the CADASIL like patients (Table 1 and Fig. 3). And recurrent ischemic events should be considered as a new risk factor in the diagnosis of CADASIL. In this research, although the major first complaints of CADASIL patients are still migraine, TIA/stroke, psychiatric disturbance and cognitive impairment as indicated in other research [6, 9, 29, 30], there are no statistical differences between CADASIL and CADASIL-like patients (Table 1). These findings support the idea that no clinical characteristics of CADASIL is pathognomonic [13]. Instead, recurrent ischemic strokes were more frequently observed in CADASIL patients than in CADASIL-like patients in our study. One possible explanation for the phenomenon is that GOM deposits in the medial layer of small penetrating arteries may promote the thickening of these vessels in CADASIL patients [31]. And any subtle changes of cerebral blood-flow dynamic or small lesions of atherosclerotic plaque, which may not be sufficient to induce stroke in CADASIL-like patients, would cause recurrent small cerebral infarcts by decreased blood perfusion [32]. Future animal experiments are needed to testify the hypothesis. Taken together, we believed that recurrent ischemic events should be considered as a new risk factor for the diagnosis of CADASIL, and be added into the CADASIL scale.

Of note, during the diagnosis of CADASIL, current opinion pays lots of attention to the early onset age of genetically inherited disease [1, 15]. For example, in CADASIL scale, TIA/Stroke < 50 years old is given a score of 2, while the recurrent ischemic events has not been taken into consideration [15]. However in our investigation, the onset ages of CADASIL were relatively older than that in the other studies conducted on Caucasian population [6, 9], and showed no difference with the onset ages in CADASIL-like patients. We speculated that one reason is migraine was less frequently observed as the first clinical manifestation in Chinese CADASIL patients than in Caucasian patients, who usually showed the symptom as early as 30 years old [1, 9]. On the other hand, the early onset of the migraine symptoms may also indicate the other potential genetic-related ischemic encephalopathies, such as mitochondrial encephalomyopathy, lactic-acidosis, stroke-like episodes (MELAS, caused by *MTTL1* mutation), familial hemiplegic migraine (FHM, caused by *CACNA1/ATP1A2/SCN1A* mutations) and Retinopathy, vascular, cerebral and renal involvement, Raynaud and migraine attacks (HERNS, caused by *TREX1* mutation) [33]. To rule out these differential diagnosis of CADASIL, next generation sequencing (NGS)-based gene panel test covering all the encephalopathy-related gene mutations may be an useful diagnostic tool to gain deeper understanding of these diseases in the future.

As an inherited single-gene disorder, it is commonly assumed that the pathological changes of CADASIL are mainly due to the effects of *NOTCH3* gene mutations, but not the effects of other factors, such as cerebral vascular risk factors in the CADASIL-like patients [6, 28]. It has been reported that, compared with CADASIL-like patients, CADASIL patients had a lower percentage of vascular risk factors [6, 8, 34]. And patients with large vessel infarctions or artherosclerosis of intracranial vessels were even excluded from the CADAIL cohort in some studies [29]. Thus, we analyzed the differences of vascular risk factors between CADASIL and CADASIL-like patients by history taking and auxiliary tests, and evaluated whether these risk factors could help in differentiating the CADASIL patients from the CADASIL-like patients. Unexpectedly, when compared the history of vascular risk factors and the biochemical items (Table 1 and Table 2) indicating the occurrence of white matter change and cerebral infarcts, there were no significant differences between CADASIL and CADASIL-like patients. Although CADASIL-like patients demonstrated higher percentage of atherosclerosis (but not significant when compared with CADASIL patients), we still noticed 50 % patients of CADASIL had stenosis of intracranial vessels and peripheral vessels, respectively. This is much higher than that reported by Yin et al. in other region of China [10]. Our data suggested that not only the genetic effects of NOTCH3 gene mutations, but also traditional vascular risk factors (or both of them) might contribute to the pathological changes during the development of CADASIL disease. In addition, in contrast to previous findings that the pathological changes of CADASIL mainly appeared in the medium sized and small arterioles and occasionally in the vein (which could not be detected in DSA imaging) [4, 12], we noticed more frequent dysplasia of the major cerebral vessels in the DSA imaging in the CADASIL patients than that in the CADASIL like patients. The data indicated that the pathological change of CADASIL could also influence the major cerebral artery in certain circumstances, and this characteristic dysplasia of major vessels in DSA may be used as a new portential risk factor for the refined CADASIL scale. Together, our data revealed that the vascular risk factors and abnormality of intracranial arteries should not be the reasons to rule out the CADASIL diagnosis. And more future researches are needed to establish a precise relationship of the CADASIL phenotype with its genotype as well as the environmental risk factors so that a personalized diagnosis strategy could be designed. We believed that direct next generation sequencing of gene panels that related to all the known encephalopathies simultaneously instead of applying CADASIL scale may be the new orientation for CADASIL diagnosis and research in the near future.

## Conclusion

In conclusion, our data indicated that the screening efficacy of CADASIL scale to identify the probands of the genetic disease varied with different populations. Recurrent cerebral ischemic events, temporal pole involvements (but not the external capsule) in MRI imaging and dysplasia of cerebral blood vessels in DSA may be the new potential risk factors for the CADASIL scale suitable for Chinese patients. Our study provides new insights into the characterization and specific diagnosis of the CADASIL disease, and indicated the importance of gene panel test for accurate diagnosis and research of a specific encephalopathy in the future.
